# A Pathway to High Quality Clinical Trials in IgA Vasculitis Nephritis: Meeting Proceedings From a Multiprofessional International Collaborative Workshop

**DOI:** 10.1016/j.ekir.2025.103729

**Published:** 2025-12-16

**Authors:** Louise Oni, Rona Smith, Seza Ozen, Chloe Williams, Elin Davies, Charlotte King, Paul Brogan, Mark Haas, Jonathan Barratt, Jeffrey Hafkin, Despina Eleftheriou, Karuna Keat, EMD Smith, Wen Ding, Chee Cheung, Caroline Platt, Evangéline Pillebout, Andrew Chetwynd, Areefa Alladin, Augusto Vaglio, Caroline Jones, Clare Pain, Cord Sunderkötter, John Peipert, Emily Barnes, Giorgio Trivioli, Hayley Hardwick, Henry Morgan, Ingeborg Bajema, James Wason, Joshua Wade, Judith Sanchez-Manubens, Kelly Vernon, Lisa Willcocks, Lorraine Harper, Lowena Lindsay, Madalina Andreea Beldie, Matko Marlais, Michelle O’Shaughnessy, Panagoula Gkargkoula, Patrick Hamilton, Reima Bakry, Roxana Bogos, Selcan Demir, Silke Brix, Simone Appenzeller, Tarun Bansal, Zoi Anastasa, Stephen David Marks, Alexandra Audemard-Verger, Thomas Renson, Marija Jelusic, David Jayne, Alan Salama

**Affiliations:** 1 Department of Renal Medicine, University College London Centre for Kidney and Bladder Health, London, UK; 2 Department of Paediatric Nephrology, Great Ormond Street Hospital, London, UK; 3 Department of Women’s and Children’s Health, University of Liverpool, Liverpool, UK; 4 Department of Medicine, University of Cambridge, Cambridge, UK; 5 Department of Renal Medicine, Cambridge University Hospital, Cambridge, UK; 6 Department of Pediatrics, Hacettepe University, Ankara, Turkey; 7 Department of Paediatrics, Northwest Deanery, Royal Manchester Children’s Hospital, Manchester, UK; 8 Department of Nephrology, Liverpool University Hospitals NHS Foundation Trust, Liverpool, UK; 9 Department of Infection, Immunology, and Rheumatology, University College London Great Ormond Street Hospital, London, UK; 10 Department of Pathology and Laboratory Medicine, Cedars-Sinai Medical Center, Los Angeles, California, USA; 11 Mayer IgA Nephropathy Laboratories, Department of Cardiovascular Sciences, University of Leicester, Leicester, UK; 12 Department of Nephrology, John Walls Renal Unit, University Hospitals of Leicester NHS Trust, Leicester, UK; 13 Otsuka Pharmaceutical Development and Commercialization, Princeton, New Jersey, USA; 14 Department of Clinical Immunology and Allergy, Campbelltown Public Hospital, New South Wales, Australia; 15 Australian and New Zealand Vasculitis Society, Melbourne, Victoria, Australia; 16 Centre for Immunobiology, School of Infection and Immunity, University of Glasgow, Glasgow, UK; 17 Department of Paediatric Rheumatology, Royal Hospital for Children, Glasgow, UK; 18 Department of Paediatric Nephrology, Bristol Royal Hospital for Children, Bristol, UK; 19 Department of Translational Health Sciences, Bristol Renal, University of Bristol, Bristol, UK; 20 Department of Nephrology, Nephrology Unit, St Louis Hospital, Paris, France; 21 Department of Biochemistry, Cell and Systems Biology, Centre for Proteome Research, Institute of Systems, Molecular and Integrative Biology, University of Liverpool, Liverpool, UK; 22 Department of Paediatric Nephrology, University of Calgary, Calgary, Alberta, Canada; 23 Department of Nephrology, Nephrology and Dialysis Unit, Meyer Children’s Hospital IRCCS, Florence, Italy; 24 Department of Biomedical, Experimental and Clinical Sciences “Mario Serio”, University of Florence, Florence, Italy; 25 Department of Paediatric Nephrology, Alder Hey Children’s NHS Foundation Trust, Liverpool, UK; 26 Department of Paediatric Rheumatology, Alder Hey Children’s NHS Foundation Trust, Liverpool, UK; 27 Department of Dermatology, University Hospital Halle, Halle, Germany; 28 MSB Medical School Berlin, Berlin, Germany; 29 Department of Applied Health Sciences, Centre for Patient Reported Outcomes Research, University of Birmingham, Birmingham, UK; 30 The University of the West of England, Bristol, UK; 31 Department of Pathology and Medical Biology, University Medical Center, University of Groningen, Groningen, The Netherlands; 32 Department of Biostatistics, Population Health Sciences Institute, Newcastle University, Newcastle Upon Tyne, UK; 33 Centre for Women's and Children's Health, Parc Taulí Hospital Universitari, Institut d’Investigació i Innovació Parc TaulíI3PT-CERCA, Sabadell, Spain; 34 Department of Pediatrics, Universitat Autònoma de Barcelona, Barcelona, Spain; 35 Department of Applied Health Sciences, University of Birmingham, Birmingham, UK; 36 Department of Paediatrics, Whiston Hospital, Merseyside, UK; 37 Department of Paediatrics, Grigore T. Popa University of Medicine and Pharmacy Iasi, Iaşi, Romania; 38 Centre for Translational Medicine, Semmelweis University, Budapest, Hungary; 39 Department of Paediatric Nephrology, “Sf. Maria” Clinical Emergency Hospital for Children, Iaşi, Romania; 40 Department of Nephrology, Galway University Hospitals, Galway, Ireland; 41 Department of Nephrology, Sussex Kidney Unit, University Hospitals of Sussex, Brighton, UK; 42 Renal, Haematology and Transplantation Unit, Manchester University NHS Foundation Trust, Manchester, UK; 43 Division of Cell Matrix Biology and Regenerative Medicine, University of Manchester, Manchester, UK; 44 Department of Pediatrics, Maternity and Children Specialized Hospital, Jeddah, Saudi Arabia; 45 Department of Pediatric Rheumatology, Eskişehir Osmangazi University, Eskişehir, Turkey; 46 Department of Orthopaedics, Rheumatology and Traumatology, School of Medical Science, University of Campinas, Campinas, Brazil; 47 Department of Nephrology, Bradford Teaching Hospitals, Bradford, UK; 48 Representative of Vasculitis UK Charity, Vasculitis International, and ERN RITA ePAG Groups, Maidstone, UK; 49 NIHR GOSH Clinical Research Facility, Great Ormond Street Hospital, London, UK; 50 Department of Internal Medicine, Tours University, Tours, France; 51 Department of Pediatric Rheumatology and Nephrology, Ghent University Hospital, Ghent, Belgium; 52 European Reference Network for Rare Immunodeficiency, Autoinflammatory and Autoimmune Diseases, Ghent University Hospital, Ghent, Belgium; 53 European Reference Network for Rare Kidney Diseases, University Hospital, Ghent, Belgium; 54 Department of Paediatrics, University Hospital Centre Zagreb, University of Zagreb School of Medicine, Zagreb, Croatia

**Keywords:** clinical trial, collaborative workshop, IgA vasculitis, nephritis

## Abstract

IgA vasculitis (IgAV) is an autoimmune disease that affects the small vessels of the skin, joints, gastrointestinal (GI) tract, and kidneys. In the long term, IgAV associated with nephritis (IgAV-N) can progress to kidney failure. Evidence-based clinical studies of IgAV-N are few, leading to huge variations in treatment approaches and suboptimal outcomes. The wealth of emerging efficacious treatments for IgA nephrology brings new opportunities to this disease. The aim of this report is to describe the proceedings of a multiprofessional collaborative workshop convened to identify the barriers to developing high quality evidence for patients with IgAV-N. A multiprofessional group consisting of 53 attendees from 13 countries met. The meeting was represented by a variety of professional backgrounds, including lay attendees, with different levels of expertise (32% professors and 19% midcareer doctors). Using predefined aims, key themes were extracted, and an action plan developed. Consensus was obtained that there is sufficient similarity between adults and children in terms of the organs involved, pathophysiology, histological features, and likely response to treatment. Important differences included the greater spontaneous improvement in children and worse kidney outcomes in some populations. It was agreed that patients at greatest risk of kidney failure should be the primary focus of initial clinical trials. Important considerations included the following: diagnostic classification for adult onset IgAV, observational data, evidence of scientific similarity to IgA nephropathy (IgAN), an age-inclusive approach to trial design, systemic disease secondary end points, and the inclusion of patient-reported outcomes. This manuscript communicates an expert-informed pathway to high-quality evidence for IgAV-N.

## Introduction

IgAV is a small vessel autoimmune vasculitis that is mediated by perivascular deposition of immune complexes containing galactose-deficient-IgA1. Although IgAV is a rare disease, it is the most common form of vasculitis seen in the pediatric population with an incidence of about 150 to 200 cases per million population and a leading cause for a pediatric nephrologist to conduct a kidney biopsy.^1^^,^^2^ In adults, it is far less common with an incidence of about 1 case per million population and there remains major gaps in identifying the true incidence of adult onset IgAV, with studies skewed toward those with major organ involvement when compared with childhood onset cohorts.^2^ For children, there are internationally recognized EULAR/PRINTO/PReS classification criteria^3^ and the European IgAV study group are currently developing adult consensus criteria. The etiology of this disease is multifactorial and geographical variation plus environmental triggers have been described.^4^ Polygenic risk variants are also reported, such as those implicated with human leucocyte antigen, cytokine mediator pathways, and the complement pathway.^5^ IgAV can be triggered by the stimulation of the immune system through acute infection or vaccinations; and mostly in adults, it may be secondary to malignancy, liver disease, or drug induced.^6^

Histologically, IgAV is identified as a small vessel leukocytoclastic vasculitis and the disease typically involves 4 organs. These organs include the skin, particularly the postcapillary venules; the joints; the GI tract; and the kidneys. In most patients, the striking lower limb cutaneous skin vasculitis is the initial symptom, and this prompts a medical review leading to a diagnosis. In the short term, skin manifestations, joint pain, and GI involvement predominate; however, a third of patients will subsequently develop nephritis (known as IgAV-N) occurring days, weeks, or months after the initial presentation.^7^ Particularly in children, the disease is a monophasic illness, that can spontaneously remit with no long-term sequalae and a positive outcome. Spontaneous remission in cases of biopsy-proven IgAV-N is less common and the condition in adolescents and adults may be more persistent.^8^^,^^9^ In the long term, kidney involvement contributes the most to morbidity, with nephritis observed in about 30% of patients during the acute phase.^4^^,^^10^ Limited observational data demonstrates that the kidney outcomes are not benign with about 20% of patients with biopsy-proven IgAV-N having significant levels of proteinuria 10 years after disease onset.^11^ The kidney outcomes correlate with age, with 1% to 3% of all children presenting with IgAV being at risk of kidney failure and this increases to 24% in the elderly.^12^ Failure to achieve complete remission of proteinuria, like other forms of glomerulonephritis, is associated with chronic kidney disease progression.^13^ Furthermore, some patients may relapse in adulthood, or evolve into IgAN with single organ involvement of only the kidney; indeed, IgAN and IgAV-N cannot be distinguished from each other on kidney biopsy. Approximately 12% of patients with IgAV-N can develop recurrence of IgA-related disease in kidney transplant, emphasizing the importance of early intervention to prevent the journey to chronic kidney disease.^14^

Patients with IgAV-N at high risk of kidney disease progression have been characterized. In a study evaluating the clinical features and kidney biopsy findings of 262 children and 99 adults, the high risk patients (defined as a risk of ≥30% decline in estimated glomerular filtration rate or developing end-stage kidney failure) had more noncutaneous manifestations, a lower estimated glomerular filtration rate at the time of kidney biopsy, were aged ≤ 18 years, or had histological presence of endocapillary hypercellularity.^15^ Similar findings have been reported in meta-analysis studies.^10^ Although defining high risk may be achievable, there remains a lack of objective measure to quantify overall disease activity because the Birmingham vasculitis activity score, and its pediatric equivalent, have limited validation in this specific form of vasculitis.^16^^,^^17^

**Figure 1:**
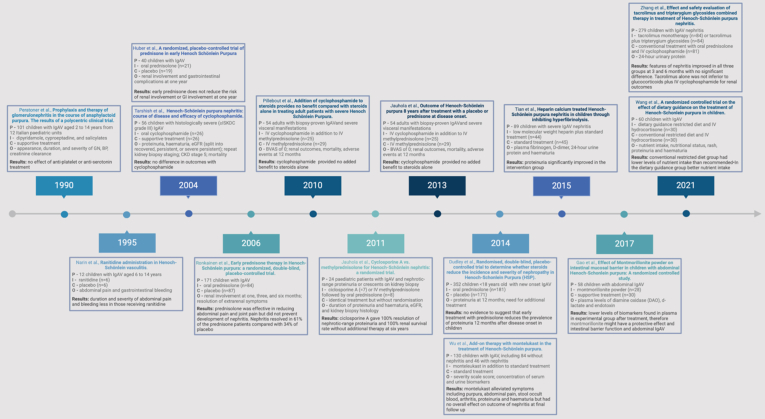
A timeline illustrating the clinical trials that have been conducted in patients with IgA vasculitis since 1990. CKD, chronic kidney disease; eGFR, estimated glomerular filtration rate; IgAV, IgA vasculitis.

For the management of nephritis, multicenter cohort studies report the frequent use of broad-spectrum immunosuppression, predominantly the use of corticosteroids and mycophenolate mofetil; however, calcineurin inhibitors and B-cell depletion have been used in both children and adults.^15^^,^^18^^,^^19^ Previous clinical trials have been small and heterogenous (Figure 1); however, there is perhaps a trend toward more targeted therapies, and an open label clinical trial in 54 adults with biopsy-proven IgAV-N demonstrated no benefit from the addition of cyclophosphamide to corticosteroids on improving Birmingham vasculitis activity score scores.^20^ As a result, Cochrane reviews have repeatedly been unable to support evidence-based recommendations for IgAV,^21^ which have led to expert consensus-based approaches, including the European Single Hub and Access Point for Paediatric Rheumatology initiative.^22^ In patients with suspected active nephritis, the indications for when to perform a kidney biopsy have been aligned in the International Pediatric Nephrology Association guidelines and the Kidney Diseases: Improving Global Outcomes glomerular diseases executive summary.^22^, ^23^, ^24^

There is a major unmet need for evidence-based treatments in IgAV-N and it is the leading cause of morbidity. Considering the recognition that the disease pathogenesis of IgAV-N shares major similarities with IgAN,^25^ such as the involvement of circulating galactose-deficient IgA1 and histological classification scores validated for IgAN perform relatively well in IgAV-N,^15^^,^^26^^,^^27^ this may provide the opportunity to reposition treatments because of the wealth of active clinical trials in IgAN.^28^ Furthermore, the visible rash seen in patients with IgAV may eventually provide a window of opportunity to intervene early, a situation not seen in many other forms of glomerulonephritis; meaning complete prevention of chronic kidney disease may even be feasible. Because of the complexities of conducting clinical trials in rare, clinically heterogeneous diseases, especially those predominating in children, changing the treatment paradigm for kidney disease secondary to IgAV requires a highly collaborative and age-inclusive approach.

#### Aim

The aim of this paper was to report the outcome of an international multiprofessional collaborative workshop to develop a pathway to drive high-quality clinical trials for patients with IgAV-N.

### Methodology

#### Objectives

The objectives of the event were as follows:

1. To determine the similarities between pediatric and adult-onset disease.
2. To explore treatments in other glomerular or vasculitic conditions that could be evaluated in IgAV-N.
3. To agree on the subgroup of patients with IgAV-N at high risk of progression to kidney failure.
4. To determine whether extrapolation, bridging biomarkers, or novel trial designs could be considered.
5. To discuss inclusive trial designs to capture all ages of patients who experience this disease.
6. To identify suitable outcome measures for treatment efficacy.

#### Participants

Participants were invited to attend through existing international networks that included vasculitis networks (UK Ireland Vasculitis Society; European IgA Vasculitis Group (i.e., EUGAVAS study group); Pediatric Rheumatology European Society vasculitis working party, Australia and New Zealand Vasculitis Society, glomerulonephritis groups (UK Glomerulonephritis Clinical Studies Group), pediatric groups (British Association for Paediatric Nephrology, European Paediatric Nephrology Association Glomerular Diseases Working Group, Paediatric Rheumatology European Society) and IgA nephropathy collaborations (International IgA Nephropathy Research Network, European Academy of Dermatology and Venereology, Task Force vasculitis and vasculopathy). Expressions of interest were open for a period of 3 months and then places were confirmed. Of the confirmed attendees, 12 participants, representing diverse personal and professional characteristics, were invited to present short summaries aligned with their area of expertise as part of the agenda. Patients were represented by specialist charity partners. All participants received reimbursement for travel, accommodation, and subsidence. Colleagues who were unable to attend the event in person were encouraged to engage and contribute before and after the event using email correspondence or virtual meetings.

#### Setting

The group of multiprofessional experts convened on February 13 and 14, 2025 at Alder Hey Children’s NHS Foundation Trust Hospital in Liverpool, UK.

#### Agenda

The agenda was divided into 2 sections with brief evidence-based presentations, interspersed with discussion. The agenda covered the natural history of the disease in both children and adults to inform similarities and opportunities to intervene during the disease course. The study population, interventions, comparators, primary and secondary outcomes, adaptations required to be age inclusive, feasibility, and power were discussed. Biobanking and additional exploratory studies were included together with the importance of patient-reported outcome measures. The event agenda concluded with exploring funding opportunities and confirmation of action points.

#### Data Extraction

Using the aims and objectives within the framework of the agenda, key themes were extracted from the event, an action plan of critical evidence gaps was developed and proposed clinical trial ideas were generated.

#### Funding and Approvals

The meeting was funded as part of the LifeArc-KRUK Translational Centre for Rare Kidney Disease project (LifeArc is a charity registered in England and Wales under no. 1015243 and in Scotland under no. SC037861; grant no. 10749) in addition to a voluntary donation provided as an independent educational grant from CSL Vifor. No formal approvals were required for this project.

## Results

#### Participants and Introduction

The 2-day event included 53 attendees, representing 13 countries across 5 continents. A total of 39 participants (74%) attended from the host country, UK and 14 (26%) were from other countries. The countries represented included UK, Ireland, USA, Canada, Australia, Turkey, France, Italy, Germany, The Netherlands, Spain, Belgium, Croatia, Romania, Hungary, Saudi Arabia, and Brazil. The professions represented included pediatric nephrology consultants, pediatric rheumatology consultants, pediatric vasculitis consultants, adult nephrology consultants, pediatric trainees, nephrology trainees, histopathologists, immunologists, industry colleagues, trialists, scientists, a dermatologist, a patient-reported outcome measurement expert, an operational manager, an administrator, and a statistician. The participants were of varying stages of their professional careers with 17 professors and 10 midcareer doctors.

#### Patient Involvement

Patient representatives stated, “It is shocking that nothing has changed in 20 years” and their engagement as patient partners was highlighted. The inclusion of patient-reported outcome measures to capture the burden of symptoms relevant to this disease was included in the discussion, using the selection of patient-reported outcome measures validated to regulatory standards, such as symptomatic edema^24^ and those capturing vasculitic features.^29^ Notably, the need for parent- or caregiver-reported measures to capture symptom and functional impacts among young children will be needed to ensure this group is captured. Patient involvement is imperative to drive the importance of evidence in this disease because the current narrative emphasizes that this disease is fully self-resolving and only occurs in children. A patient-led awareness campaign may help to change this perception.

#### Childhood and Adult-onset IgAV Disease

The threshold where professionals would regard the degree of similarity in the disease and the expected response to treatment between 2 groups (children and adults) was discussed. There is no specific threshold for sufficient similarity; however, 70% is often considered adequate when using modified Delphi techniques.^30^ The experts felt that there was sufficient similarity related to the organs involved; and specifically, skin manifestations across the pediatric and adult populations. The group agreed that the pathophysiology, and therefore the mechanisms of action of treatments, are likely to be sufficiently similar for all ages, while appreciating that diagnosis in adults may be more challenging in the absence of consensus classification criteria. The histopathological features in IgAV-N across children and adults were believed to be sufficiently similar.^15^ However, additional clinical features, the disease course, and kidney failure outcomes were not deemed to be sufficiently similar between the age groups, and these elements would need to be considered when designing a trial.

#### IgAV-N and Other Glomerulonephritides

The kidney histology in patients with IgAV-N when compared with the histology of IgAN were deemed to be highly similar. It was noted that endocapillary hypercellularity is the most important histologic feature with regard to clinical outcomes in IgAV-N.^15^^,^^31^ Therefore, the Oxford MEST-C classification, developed and validated for IgAN in adults and children, appears the most suitable for histological scoring in IgAV-N when compared with the International Study of Kidney Diseases in Children classification, which does not include endocapillary hypercellularity.^31^ The use of the Oxford MEST-C scoring system in this condition has the added benefit of providing consistency with trial protocols in IgAN. Glomerular deposits of complement C3 and their colocalization with IgA were recognized to correlate with disease severity in both IgAN and IgAV-N.^32^ Therefore, drugs targeting complement pathways and especially the alternative pathway, were felt to be promising because of the biological similarities and proven efficacy in other forms of glomerulonephritis. Patients going into spontaneous remission was expressed as a trial design challenge. Screening patients from the time of diagnosis and ensuring ≥2 consecutive elevated urine proteinuria measurements before entry may be a feasible screening method to exclude those who may naturally remit early. It was also recognized that fewer patients remit by the time they undergo a kidney biopsy and using this high-risk group to begin to accelerate trials in this disease may be the most successful option. Enriching the natural history data to better describe IgAV-N would inform the precise population and guide clinical trial design, and hopefully act as an incentive to support industry collaborations, as was demonstrated by the impactful long-term data obtained in IgAN.^33^ Observational data so far suggests that the current standard-of-care for immunosuppressive treatments is most frequently corticosteroids and mycophenolate mofetil.^18^ Other considerations included establishing the long-term cost implications of IgAV-N, which largely affects younger individuals and how chronic kidney disease progression is costly over the life course. A formal health economic analysis would justify the treatment costs and help secure successful implementation of treatments into clinical care following evidence generation.

#### Defining the Population at High Risk of Kidney Progression Because of IgAV

The working group agreed that gaining evidence to guide the management of nephritis was a priority for patients with this condition, with active nephritis being defined by increased proteinuria, with or without impaired kidney function and/or biopsy-proven inflammation. In terms of other organ involvement, GI involvement was felt to be the second priority, followed by cutaneous involvement, then joint involvement, which is often self-limiting. With regard to early markers of kidney disease, isolated microscopic hematuria was felt to be of unknown significance. Reducing the variability on when to perform a kidney biopsy was highlighted, with attendees suggesting that a urine protein-to-creatinine ratio value ranging between 100 and 500 mg/mmol Cr would be suitable inclusion criterion. The recent International Pediatric Nephrology Association guidelines, which outline kidney biopsy indications with a urine protein-to-creatinine ratio > 200 mg/mmol Cr and/or a reduced estimated glomerular filtration rate < 90 ml/min per 1.73 m^2^ attributable to active disease, moderate proteinuria of 100 to 200 mg/mmol Cr if present for > 2 weeks and urine protein-to-creatinine ratio is 20 to 100 mg/mmol Cr for 4 to 12 weeks, if there has been no improvement after renin angiotensin-aldosterone system inhibition, may act as a suitable benchmark.^24^

Attendees acknowledged that disease scoring using the Birmingham vasculitis activity score and pediatric equivalent has previously formed part of composite outcome measure in trials for other forms of vasculitis that have aided drug licensing approvals and could be considered. There was recognition of the need to validate these tools, or adjust them to be more disease-specific, because the validation of Birmingham vasculitis activity score (version 3) only included 10 adults with IgAV (3.2%)^34^ and only 1 child (1.6%) was included in the validation of the pediatric equivalent despite it being the most common form of vasculitis in children.^17^ Preliminary creation of a disease-specific scoring tool “The IgA-VAS,” to support better phenotyping and/or objectively measure outcomes, was presented and this would require further refinement and validation in a fully representative cohort. This could be achieved by forming an international expert consensus panel and validation patient cohorts to mature the preliminary tool.

#### Extrapolation, Bridging Biomarkers, Scientific Markers, and Novel Trial Designs

Overall, a phase 2 proof of concept study would ensure feasibility of conducting trials in this population to prepare for confirmatory phase 3 clinical trials. This may be best achieved by using the current regulatory approved end points for nephrology trials, including proteinuria reduction and change in estimated glomerular filtration rate as primary outcome measures. A protocol would require safety and drug kinetics that are relevant for all ages. Generating label-enabling data were reported to be an incentive for industry partners.

Novel trial designs, including the use of Bayesian approaches to incorporate external data were discussed. A multiarm platform trial design, that consists of a master protocol where multiple trial interventions can be evaluated more efficiently, was seen as a superior way to establish a perpetual framework to evaluate treatments within a shorter time frame and to enable evaluation of the wealth of treatments evolving for IgAN.^35^ The trial templates and progression of evidence used for other rare kidney diseases, such as C3 glomerulopathy and IgAN, could provide rich learning; and exemplars integrating genetic stratification are emerging.^36^^,^^37^ Consideration of how to define and manage a disease flare during a trial would need to be included. The group felt that combining adult patients with IgAV-N into IgAN studies, perhaps using an umbrella design, was beginning to be used in a few studies; however, there were concerns about recruitment numbers for adults with IgAV-N and whether it may hinder the progress being made in IgAN. In contrast, in the pediatric population, where many of the agents have not yet been evaluated in children with IgAN, it was felt that there could be an opportunity to merge IgAN and IgAV-N although this would need further consideration.

#### Considerations to Ensure Feasibility of a Clinical Trial in IgAV

The group agreed that the medications needed to be in a suitable formulation for administration to patients of all ages. This means that some agents, such as the targeted-release formulation of budesonide (nefecon), may not be appropriate for evaluation across all ages because they are modelled on the size of an adult GI tract. It was felt that regulators would need to be satisfied that the 2 diseases, IgAV-N and IgAN, were sufficiently scientifically similar to justify the evaluation of similar treatments. Ongoing research to explore the biological similarities is therefore encouraged and a subsequent survey after this event has demonstrated that 76.5% of the Renal Pathology Society members would prefer to report a kidney biopsy for IgAV-N using the Oxford MEST-C classification. Potential future therapeutic agents were discussed, and these are presented in detail in Table 1.^38^, ^39^, ^40^, ^41^, ^42^, ^43^, ^44^, ^45^, ^46^, ^47^, ^48^, ^49^, ^50^, ^51^, ^52^, ^53^, ^54^, ^55^, ^56^, ^57^, ^58^, ^59^, ^60^, ^61^, ^62^, ^63^, ^64^, ^65^, ^66^, ^67^, ^68^, ^69^ There were collective desires to investigate novel biomarkers, such as galactose-deficient IgA or B-cell repertoire, to better stratify patients as exploratory components in a trial.

**Table 1:** A list of potential pipeline therapeutic agents from the literature that could be considered in IgAV-N, grouped according to mechanism of action

Agent	Therapeutic subclass	Supporting comments and evidence in IgAV, IgAN or other forms of vasculitis
Targeting B-cell pathways		
Rituximab	Depletes CD20 cells, chimeric formulation	Case series in IgAV^19^^,^^38^^,^^39^ RIGA trial underway in adults with IgAV. AAV induction and maintenance.^40^
Obinutuzumab	Depletes CD20 cells, fully humanized formulation	Deeper B-cell depletion than rituximab, evaluated in several diseases including lupus nephritis,^41^ membranous nephropathy,^42^ ANCA-associated vasculitis^43^ and childhood nephrotic syndrome.^44^
Sibeprenlimab	Inhibits APRIL	ENVISION phase 2 trial with VISIONARY phase 3 trial in IgAN awaiting completion.^45^
Zigakibart	Inhibits APRIL	BEYOND phase 3 trial in IgAN awaiting outcome.^46^
Atacicept	Recombinant fusion protein binds BAFF and APRIL	ORIGIN Phase 2b trial in IgAN reduced proteinuria with a positive open label extension study and phase 3 awaiting completion.^47^^,^^48^ PIONEER trial is underway and includes adults and adolescents with a range of glomerular diseases including IgAV-N.^49^
Telitacicept	Inhibits BAFF and APRIL	Phase 2 trial clinically meaningful reduction in proteinuria in IgAN. Awaiting phase 3 trial results^50^ and pediatric study is underway.^51^
Povetacicept	Inhibits BAFF and APRIL	Awaiting phase 1 / 2 trial results in IgAN, membranous nephropathy, lupus nephritis and antineutrophil cytoplasmic autoantibody (ANCA)-associated vasculitis.^52^ RAINER phase 3 trial in IgAN awaiting completion.
Bortezomib	Depletes plasma B cells	Case studies in IgAV and IgAN^53^^,^^54^
Daratumumab	Targets CD38	Trials conducted in multiple glomerular diseases with early promising results.^55^
Targeting complement pathways		
Avacopan	Complement 5a receptor inhibitor	Completed pilot phase 2 in IgAN^56^ and successful trial in ANCA vasculitis.^57^
Pegcetacoplan	Complement 3 factor inhibitor	Phase 2 trial in C3 glomerulopathy and phase 3 findings awaited^58^
Iptacopan	Complement factor B inhibitor	Reduction in proteinuria seen in the phase 3 APPLAUSE-IgAN trial^59^ and phase 3 trials in C3 glomerulopathy have successfully met end points.^60^^,^^61^
Cemdisiran	Complement 5 factor inhibitor	Completed phase 2 trial in IgAN with reduction in proteinuria.^62^
Ravulizumab	Terminal pathway activation, complement 5 factor inhibitor	Phase 2 clinical SANCTUARY Study demonstrates early sustained improvements in proteinuria in IgAN.^63^ ICAN Phase 3 trial awaiting completion includes patients with IgAN and inactive IgAV-N. Pediatric IgAN study underway.^64^
Sefaxersen (IONIS -FB-LRx)	Antisense oligonucleotide inhibitor of complement factor B.	Early phase trials positive with progression to phase 2 and 3 (IMAGINATION) trials underway for IgAN.^65^
Narsoplimab	MASP-2 inhibitor	ARTERMIS-IgAN phase 3 trial terminated at interim analysis due to lack of efficacy in IgAN.
Targeting other pathways		
Sparsentan	Dual endothelin and angiotensin receptor antagonist	PROTECT trial in IgAN demonstrated efficacy,^66^ phase 2 pediatric study on glomerular proteinuria (EPPIK) underway and includes patients with IgAV.
Atrasentan	Selective endothelin A receptor inhibitor	ALIGN trial in IgAN is underway^67^
Tocilizumab	IL-6 receptor blocker	Case report in IgAV,^68^ used in other types of rheumatological conditions.
Targeted release budesonide	Glucocorticoid that specifically targets the gut associated lymphoid tissue of the small intestine.	Trials in IgAN show reduction of proteinuria as add-on to standard of care.^69^ Formulation not suitable for pediatric GI tract and uncertain origin of Gd-IgA in IgAV.

Gd-IgA; galactose-deficient-IgA1; IgAN, IgA nephropathy; IgAV, IgA vasculitis; IgAV-N, IgAV associated with nephritis.

### Conclusion

Despite major recent advances in other forms of glomerulonephritis, particularly IgAN, IgAV-N is a highly neglected rare disease in terms of evidence-based therapeutic options. This manuscript reports the proceedings of a multiprofessional, collaborative workshop of international experts to develop collective solutions toward generating high-quality evidence for patients with IgAV-N. The global attendees each brought a unique perspective and a shared vision of creating a pipeline of age-inclusive, high-quality clinical trials starting with feasible phase 2 studies to facilitate progression into confirmatory phase 3 studies. The key action points generated from the meeting are outlined in Table 2; and through collaborative efforts, they are being completed to achieve this ambition. Overall, there was an overwhelming desire to change the paradigm for this condition, and this manuscript serves as a benchmark toward establishing a comprehensive path to generating high-quality evidence for patients with IgAV-N.

**Table 2:** A summary of the key priorities to support progression toward high quality clinical trials for IgAV-N

Key components	Actions
Enhancing data and aligning clinical care	
Observational cohort data	To enrich the published literature using established registries from multiple sources.
Disease specific scoring tool to objectively phenotype patients.	To define and undertake the methodology to improve and achieve validation of a disease specific vasculitis activity scoring tool for IgAV.
Define the cost of managing patients with IgAV.	Develop the methodology to conduct a formal health economic analysis of this condition.
Align clinical care	Promote the use of internationally agreed clinical practice guidelines.
Improving the scientific understanding	
Demonstrate biological similarity with IgAN.	Use multiomic techniques to evaluate the granular details between the diseases, for example spatial transcriptomics and proteomics of the kidney biopsy tissue.
Understand and compare the sites of production of galactose-deficient IgA1 (Gd-IgA1) in IgAN and IgAV in adults and children.	Conduct mechanistic studies to determine the origin of Gd-IgA1 and its connection with potential triggers such as epipharynx-kidney axis.
Evaluate whether Gd-IgA1 or other markers such as complement pathway products relate to disease activity	Evaluate complement deposition in biopsy tissue and complement components in urine and correlate these with histologic (e.g., Oxford M, E, C scores) and clinical (e.g., proteinuria, hematuria, eGFR slope) markers of disease activity and progression.
Pathological classification	Work with international networks (e.g., Renal Pathology Society, International IgA Nephropathy Network Research Group, IPNA, KDIGO) to establish a formal statement on the best practice in reporting and classifying the kidney histological features in this disease.
Progression to clinical trials	
Strengthen industry partnerships	Invest in events related to this condition to develop a positive research environment for collaboration.
Suitable agents	Create criteria to select potential agents to evaluate in this condition.
Securing funding for a clinical trial	Experts to design trial protocols and seek country specific funding opportunities using a mirrored approach to avoid duplication.
Regulatory approved end points and trial design	To design a draft trial protocol using expert input and establish international regulatory approval particularly for the end point selection.
People	
Patient engagement	To work with patient associations to highlight the lack of progress in improving this disease, the poor outcomes reported, and adult-onset disease to enhance the need for evidence. Encourage country specific patient association groups to unite internationally.
Determine the most appropriate patient-centered outcomes and end points	In partnership with patients and parents/caregivers to elicit the most important symptoms and functional impacts of IgAV in a life course approach that may be addressed with treatment in clinical research and use the results to identify or develop appropriate patient- or observer-reported outcome measures.
Engaged expert community	Invite attendees to form an international clinical trial working group for the acceleration of IgAV evidence.

eGFR, estimated glomerular filtration rate; Gd-IgA1, galactose-deficient-IgA1; IgAV, IgA vasculitis; IgAV-N, IgAV associated with nephritis; KDIGO, Kidney Disease: Improving Global Outcomes.

## Disclosure

LO provides expert consultancy for Aurinia Pharmaceuticals, Eloxx, Dimerix, Biohaven Pharmaceuticals Inc, Santhera Pharmaceuticals, Hansa biopharm, Boehringer-Ingelheim, Sandoz, Biocryst pharmaceuticals Ltd, Novartis, Bedrock, Chinook therapeutics, Roche products Ltd, Travere therapeutics Ltd, and Alentis therapeutics. SRB has received honoraria, Speakers Bureau, and Educational Grants from Vifor and Otsuka. CS has received honoraria and advisory board work for GSK and Vifor. MH has received consulting fees from Novartis, AstraZeneca, Argenx, Otsuka, Biogen, Travere, and Vera Therapeutics; and has received research funding from Argenx. LH has received honorarium from GSK, CSL Vifor, and AZ; and research funding from Vifor and MSD. WYD has equity in Purespring Therapeutics. DJ has received consultancy fees and honoraria from Alentis, Amgen, AstraZeneca, Aurinia, Boehringer, GSK, Novartis, Otsuka, Roche/Genentech, Takeda, and CSL Vifor; and research grants from GSK, Roche/Genentech, and CSL Vifor. JW has received PhD funding via the Cambridge-GSK Translational Immunology Collaboration (CG-TIC). CKC reports consulting or speaker fees from Alexion, Boehringer Ingelheim, Calliditas, CSL Vifor, Dimerix, Emerald Clinical, Novartis, Otsuka Pharmaceutical, Roche, Sobi, Stada, Travere Therapeutics, Vera Therapeutics, and Vertex; and research funding from Travere Therapeutics. ZA works for Vasculitis UK and has received honorarium and small payments for contributing in committees or reviewing materials from CSL Vifor and NOVARTIS. All the other authors declared no competing interests.
